# Divergence of canonical danger signals: The genome-level expression patterns of human mononuclear cells subjected to heat shock or lipopolysaccharide

**DOI:** 10.1186/1471-2172-9-24

**Published:** 2008-05-30

**Authors:** Hector R Wong, Kelli Odoms, Bhuvaneswari Sakthivel

**Affiliations:** 1 Department of Pediatrics, University of Cincinnati College of Medicine, Division of Critical Care Medicine, Cincinnati Children's Hospital Medical Center and Cincinnati Children's Research Foundation, Cincinnati, Ohio, USA; 2 Department of Pediatrics, University of Cincinnati College of Medicine, Division of Biomedical Informatics, Cincinnati Children's Hospital Medical Center and Cincinnati Children's Research Foundation, Cincinnati, Ohio, USA

## Abstract

**Background:**

Peripheral blood mononuclear cells (PBMC) serve a sentinel role allowing the host to efficiently sense and adapt to the presence of danger signals. Herein we have directly compared the genome-level expression patterns (microarray) of a human PBMC model (THP-1 cells) subjected to one of two canonical danger signals, heat shock or lipopolysaccharide (LPS).

**Results and Discussion:**

Based on sequential expression and statistical filters, and in comparison to control cells, we found that 3,988 genes were differentially regulated in THP-1 cells subjected to LPS stress, and 2,921 genes were differentially regulated in THP-1 cells subjected to heat shock stress. Venn analyses demonstrated that the majority of differentially regulated genes (≥ 70%) were uniquely expressed in response to one of the two danger signals. Functional analyses demonstrated that the two danger signals induced expression or repression of genes corresponding to unique pathways, molecular functions, biological processes, and gene networks. In contrast, there were 184 genes that were commonly upregulated by both stress signals, and 430 genes that were commonly downregulated by both stress signals. Interestingly, the 184 commonly upregulated genes corresponded to a gene network broadly related to inflammation, and more specifically to chemokine signaling.

**Conclusion:**

These data demonstrate that the mononuclear cell responses to the canonical stress signals, heat shock and LPS, are highly divergent. However, there is a heretofore unrecognized common pattern of gene network expression corresponding to chemokine-related biology. The data also serve as a reference database for investigators in the field of stress signaling.

## Background

Peripheral blood mononuclear cells serve a sentinel role by allowing rapid host adaptations to danger signals associated with various biological stresses such as infection, trauma, or drastic environmental changes [1]. Two of the more primitive and universal danger signals recognized by peripheral blood mononuclear cells, as well as other types of cells, are lipopolysaccharide (LPS) of gram negative bacteria and heat stress (a.k.a. heat shock). Previous studies in our laboratory, and that of others, have implied that the cellular responses to LPS and heat shock are mutually exclusive, particularly at the transcriptional level [2-6]. The transcriptional response to LPS has been traditionally viewed as one involving expression of immune- and inflammation-associated genes that serve to rid the host of infection. In contrast, the transcriptional response to heat shock has been traditionally viewed as a specific re-prioritization of gene expression toward "stress proteins" while other forms of gene expression are globally suppressed.

These two traditional paradigms involving the transcriptional responses to LPS or heat stress may represent oversimplifications. For example, transcriptional profiling experiments have suggested the existence of previously unrecognized genes that are expressed in response to LPS stimulation [7-12]. Our own studies have demonstrated that genes not traditionally regarded as "heat shock proteins" can be specifically regulated and expressed in response to heat shock [13-18].

In the current work, we sought to expand our understanding of the peripheral blood mononuclear cell transcriptional response to canonical stress/danger signals. We have exposed peripheral blood mononuclear cells to either LPS or heat shock, and have directly compared their respective genome-level responses using microarray technology. The focus of the analytical approach is coordinated expression/repression of genes corresponding to functional annotations and gene networks.

## Methods

### Cell model and experimental conditions

Human THP-1 mononuclear cells (American Type Culture Collection, Manassas, VA; ATCC#: TIB-202™) were used as a model of peripheral blood mononuclear cells as previously described [19]. Cells were maintained in a room air/5% CO_2 _incubator at 37°C using RPMI (Invitrogen, Carlsbad, CA) supplemented with 10% fetal bovine serum. One group of cells was exposed to LPS (1 μg/ml, *E. coli *serotype 055:B5, Sigma, St. Louis, MO) for 4 hours at 37°C. Heat shock was carried out by placing one group of cells in a dedicated room air/5% CO_2 _incubator set at 43°C for 1 hour. After heat shock, cells were returned to a 37°C, room air/5% CO_2 _incubator and allowed to recover for 4 hours. Control cells were maintained in basal growth media at 37°C. After exposure to the experimental conditions, cells were harvested for total RNA extraction as described below.

### RNA extraction and microarray hybridization

The data and protocols described in this manuscript have been deposited in the NCBI Gene Expression Omnibus (GEO, [20]) and are accessible through GEO Series accession number GSE9916.

Total RNA was isolated from THP-1 cells exposed to the above experimental conditions using the Trizol reagent (Invitrogen) according to the manufacturer's specifications. Microarray hybridization was performed by the Affymetrix Gene Chip Core facility at Cincinnati Children's Research Foundation as previously described using the Human Genome U133 Plus 2.0 GeneChip (Affymetrix, Santa Clara, CA) [21,22].

Analyses were performed using one experimental condition per chip, with each experimental condition carried out in triplicate (i.e. 3 biological replicates per experimental condition for a total of 9 individual chips). Image files were captured using an Affymetrix GeneChip Scanner 3000. CEL files were subsequently preprocessed using Robust Multiple-Array Average (RMA) normalization and GeneSpring GX 7.3 software (Agilent Technologies, Palo Alto, CA). All signal intensity-based data were used after RMA normalization, which specifically suppresses all but significant variation among lower intensity probe sets [23]. All chips were then normalized to the respective median values of the control condition.

### Analysis of microarray data

Differences in mRNA abundance between LPS-stimulated cells and control cells, and between heat shock-treated cells and control cells were determined using GeneSpring GX 7.3 (Agilent Technologies). All statistical analyses used corrections for multiple comparisons. A list of genes differentially regulated between LPS-treated cells and control cells was generated using sequential expression and statistical filters as follows. Starting with all gene probes within the microarray (54,681 gene probes) we selected the genes having ≥ 2-fold expression difference (increased or decreased expression) between the respective median values of LPS-treated cells and control cells. The genes passing this expression filter were then subjected to a statistical test (ANOVA, Benjamini-Hochberg false discovery rate of 5%) using LPS-treated cells and control cells as the comparison groups. A list of genes differentially regulated between heat shock-treated cells and control cells was generated using identical sequential expression and statistical filters, but using heat shock-treated cells and control cells as the comparison groups.

### Functional annotation analysis

Gene lists of differentially expressed genes were primarily analyzed for functional annotation enrichment using the PANTHER (Protein Analysis Through Evolutionary Relationships) Classification System [24,25]. The PANTHER Classification System is a publicly accessible relational database of functional gene annotations and is based on the existing biomedical literature. The application uses specific approaches to estimate significance (p values) based on non-redundant representations of the microarray chip and to convert the uploaded gene lists to gene lists containing a single value per gene. All PANTHER-derived data are corrected for multiple comparisons via Bonferroni.

### Derivation of gene networks

Gene lists of differentially expressed genes were also analyzed for the presence of gene networks using the Ingenuity Pathways Analysis application (IPA, Ingenuity Systems, Redwood City, CA) [26]. The IPA application provides a tool for discovery of gene networks within the uploaded gene lists. The IPA application maps each gene identifier to its corresponding gene object in the IPA Knowledge Base. These focus genes are then overlaid onto a global molecular network developed from information contained in the Knowledge Base. Networks of these focus genes are then algorithmically generated based on their connectivity. Each individual IPA network has a maximum of 35 focus genes and is assigned a significance score (based on p value) representing the likelihood that the focus genes within the network are found therein by random chance. A high number of focus genes within a dataset leads to a higher network score (equal to the negative exponent of the respective p value such that a score of 3 corresponds to a p value of 10E-3). Each of the IPA-derived gene networks provided in the Results section has a significance score of ≥ 35 (i.e. p value ≥ 10E-35) and the gene networks are presented as merges of the top two scoring networks within each of the respective gene lists.

### Derivation of gene network functional annotations

The functional annotations associated with the gene networks presented in Results section were generated using a relational database independent of the IPA application and the PANTHER database. The gene lists corresponding to the gene networks were analyzed using D.A.V.I.D. (Database for Annotation, Visualization and Integrated Discovery), which also allows public access to relational databases of functional gene annotations [27]. In the D.A.V.I.D. analytical output, "category" refers to the original database or resource from which the annotations are derived, and "term" refers to the enriched annotation terms associated with the given gene list. D.A.V.I.D is also based on the established biomedical literature and also uses specific approaches to estimate significance (p values) based on non-redundant representations of the microarray chip and to convert the uploaded gene lists to gene lists containing a single value per gene. The p values for a given category and term provide an estimate of the likelihood that a given annotation in enriched in a given gene list by chance alone. All of the p values presented in the D.A.V.I.D.-based analyses have been corrected for multiple comparisons using the Benjamini-Hochberg false discovery rate of 5%.

## Results

### Comparison of the genomic expression profiles of THP-1 cells exposed to either LPS or heat shock

Based on the filtering strategy described in the Methods section, there were 3,968 gene differentially expressed in THP-1 cells exposed to LPS. Of these 3,968 genes, 1,345 were upregulated and 2,623 were downregulated relative to control cells (see Additional file 1). In comparison, there were 2,921 genes differentially expressed in THP-1 cells exposed to heat shock. Of these 2,921 genes, 1,476 were upregulated and 1,445 were downregulated relative to control cells (see Additional file 2).

**Figure 1:**
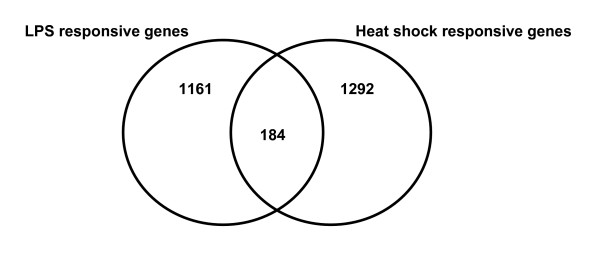
**Venn analysis comparing the 1,345 upregulated LPS-responsive genes and the 1,476 upregulated heat shock-responsive genes.** See text for gene filtering strategy.

Figure 1 depicts a Venn analysis comparing the genes upregulated in response to LPS and heat shock, respectively. Eighty-six percent of the upregulated LPS-responsive genes were uniquely expressed in response to LPS, whereas 88% of the upregulated heat shock-responsive genes were uniquely expressed in response to heat shock. In contrast, 184 upregulated genes were common to both the LPS response and the heat shock response.

**Figure 2:**
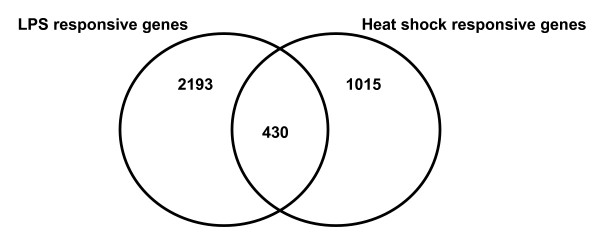
**Venn analysis comparing the 2,623 downregulated LPS-responsive genes and the 1,445 downregulated heat shock-responsive genes.** See text for gene filtering strategy.

Figure 2 depicts a Venn analysis comparing the genes downregulated in response to LPS and heat shock, respectively. Eighty-four percent of the downregulated LPS-responsive genes were uniquely repressed in response to LPS, whereas 70% of the downregulated heat shock-responsive genes were uniquely repressed in response to heat shock. In contrast, 430 downregulated genes were common to both the LPS response and the heat shock response.

Collectively, these data demonstrate that a large proportion of genes differentially expressed in response to LPS or heat shock are unique relative to the specific stress signal. However, the two stress signals also share a relatively smaller proportion of commonly regulated genes.

### Functional annotations corresponding to the upregulated LPS- and heat shock-responsive genes

To begin deriving biological meaning from the individual lists of upregulated LPS- and heat shock-responsive genes, we uploaded the respective upregulated gene lists to the PANTHER database and focused the analysis on enrichment for signaling pathways, biological processes, and molecular functions. As shown in Table 1, the upregulated LPS-responsive genes predominantly corresponded to several annotations related to inflammation, immunity, and signal transduction. In contrast, the upregulated heat shock-responsive genes predominantly corresponded to several annotations related to the stress response, molecular chaperones, and protein folding. Both upregulated gene lists corresponded to signaling pathways related to apoptosis. These data demonstrate that the individual upregulated gene lists of LPS- and heat shock-responsive genes are significantly enriched for unique functional annotations.

**Table 1:** Functional annotations associated with the upregulated LPS- and heat shock-responsive genes, respectively.

	**LPS**	**Heat Shock**
**Pathway (p value)**	• Apoptosis signaling (1.5E-10)	• Apoptosis signaling (1.8E-3)
	• Inflammation mediated by chemokine and cytokine signaling (2.1E-7)	• Angiogenesis (3.2E-2)
	• Toll receptor signaling (9.6E-6)	
	• Integrin signaling (1.4E-2)	

**Biological process (p value)**	• Immunity and defense (2.2E-25)	• Protein folding (2.1E-14)
	• Signal transduction (5.8E-23)	• Protein metabolism and modification (1.7E-10)
	• Intracellular signaling cascade (3.0E-16)	• Stress response (1.8E-10)
	• Cell surface receptor mediated signaling (2.7E-10)	• Protein phophorylation (1.7E-6)
	• Cytokine and chemokine mediated signaling (3.1E-10)	• Immunity and defense (2.0E-5)

**Molecular function (p value)**	• Signaling molecule (1.4E-10)	• Chaperone (1.2E-16)
	• Other transcription factor (3.0E-7)	• Hsp 70 family chaperone (1.9E-7)
	• Chemokine (3.3E-6)	• Protein kinase (2.8E-6)
	• Cytokine receptor (1.2E-5)	• Transcription factor (2.0E-4)
	• Select regulatory molecule (8.9E-5)	• Chaperonin (1.2E-3)

Data are based on the PANTHER database using the default parameters in the database and Bonferroni correction for multiple comparisons.

### Functional annotations corresponding to the downregulated LPS- and heat shock-responsive genes

To begin deriving biological meaning from the individual lists of downregulated LPS- and heat shock-responsive genes, we uploaded the respective downregulated gene lists to the PANTHER database and again focused the analysis on enrichment for signaling pathways, biological processes, and molecular functions. As shown in Table 2, the downregulated LPS-responsive genes predominantly corresponded to several annotations related to protein/carbohydrate metabolism, amino acid metabolism, and other biochemical processes (e.g. oxidoreductase, dehydrogenase, and lyase activity). In contrast, the downregulated heat shock-responsive genes predominantly corresponded to several annotations related to transcription and nucleotide processing. There were no common functional annotations between the downregulated LPS- and heat shock-responsive genes. These data demonstrate that the individual downregulated gene lists of LPS- and heat shock-responsive genes are significantly enriched for unique functional annotations.

**Table 2:** Functional annotations associated with the downregulated LPS- and heat shock-responsive genes, respectively.

	**LPS**	**Heat Shock**
**Pathways**	• None signficant	• None significant
**Biological process (p value)**	• Protein metabolism and modification (6.8E-7)	• Nucleoside, nucleotide and nucleic acid metabolism (1.3E-3)
	• Amino acid metabolism (5.2E-6)	• mRNA transcription regulation
	• Other metabolism (1.4E-5)	• (2.7E-3)
	• Carbohydrate metabolism (1.3E-4)	• mRNA transcription (3.3E-2)
	• Protein modification (2.2E-4)	
**Molecular function (p value)**	• Oxidoreductase (5.0E-7)	• Transferase (1.4E-3)
	• Dehydrogenase (4.4E-6)	• Transcription factor (1.9E-3)
	• Synthase and synthetase (3.3E-4)	• Nucleic acid binding (9.5E-3)
	• Transferase (5.5E-4)	
	• Lyase (1.5E-3)	

Data are based on the PANTHER database using the default parameters in the database and Bonferroni correction for multiple comparisons.

### Gene network expression corresponding to the upregulated LPS- and heat shock-responsive genes

**Figure 3:**
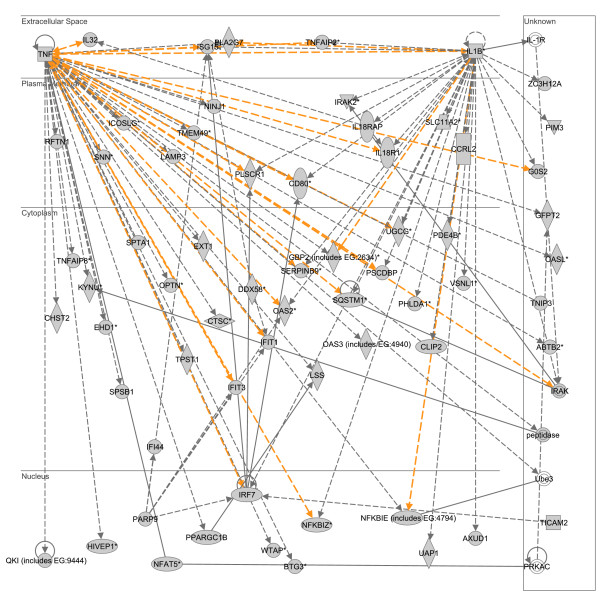
**Merge of two top scoring IPA networks derived from the list of 1,345 upregulated LPS-responsive genes.** The merged network is depicted in the context of cellular compartments. This upregulated LPS-responsive network contains two nodes in the extracellular compartment having a high degree of connectivity to other network genes: tumor necrosis factor (TNF) and interleukin-1β (IL1B). Gold lines indicate connectivity between the two merged networks. See text for network derivation, and see Additional file 3 for network gene list and Additional file 4 for network legend.

To derive further biological meaning from the individual lists of upregulated LPS- and heat shock-responsive genes, we uploaded the respective upregulated gene lists to the IPA application and focused the analysis on gene network expression. The two highest scoring, upregulated LPS-responsive gene networks were merged as depicted in Figure 3. This upregulated LPS-responsive gene network is dominated by two gene nodes, located in the extracellular compartment, and having the highest degree of connectivity relative to the other gene nodes in the network: interleukin-1β (IL1B) and tumor necrosis factor (TNF). The list of genes corresponding to this upregulated LPS-responsive gene network was subsequently uploaded to an independent database (D.A.V.I.D.) to derive the functional annotations corresponding to the gene network. The results of the D.A.V.I.D.-based analysis are provided in Table 3 and demonstrate that this upregulated LPS-responsive gene network is enriched for functional annotations corresponding to immunity and inflammation.

**Table 3:** Top 10 functional annotations for two merged networks upregulated in response to LPS (based on p value).

**Category**	**Term**	**# of genes**	**p-value**
SP_PIR_KEYWORDS	interferon induction	9	9.3E-11
GOTERM_BP_ALL	response to biotic stimulus	25	1.6E-8
GOTERM_BP_ALL	immune response	23	3.2E-8
GOTERM_BP_ALL	defense response	23	1.5E-7
GOTERM_BP_ALL	response to stimulus	28	8.4E-5
GOTERM_BP_ALL	response to stress	18	1.3E-4
GOTERM_BP_ALL	response to external stimulus	13	1.5E-4
GOTERM_BP_ALL	organismal physiological process	28	1.8E-4
GOTERM_BP_ALL	response to pest/pathogen/parasite	13	2.9E-4
GOTERM_BP_ALL	response to other organism	13	4.8E-4

The analysis is based on the default parameters in D.A.V.I.D. and uses Benjamini-Hochberg correction for multiple testing.

**Figure 4:**
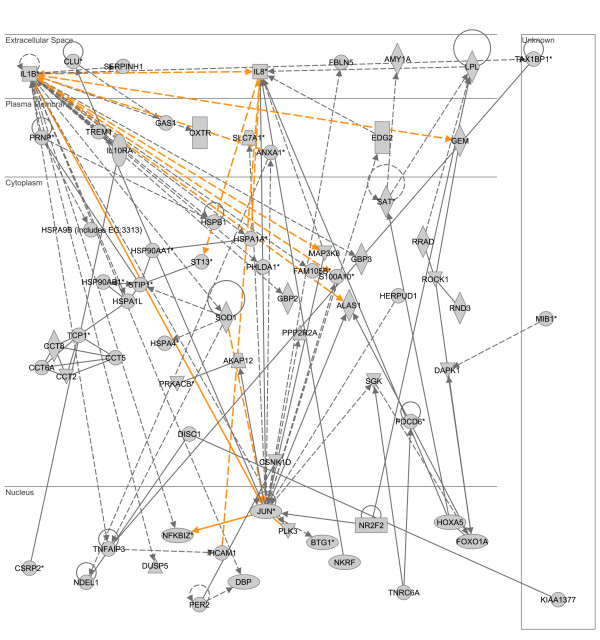
**Merge of two top scoring IPA networks derived from the list of 1,476 upregulated heat shock-responsive genes.** The merged network is depicted in the context of cellular compartments. This upregulated heat shock-responsive network contains two nodes having a high degree of connectivity to other network genes: interleukin-1β (IL1B) located in the extracellular compartment and jun oncogene (JUN) located in the nuclear compartment. Gold lines indicate connectivity between the two merged networks. See text for network derivation, and see Additional file 5 for network gene list and Additional file 4 for network legend.

The two highest scoring, upregulated heat shock-responsive gene networks were merged and analyzed in a similar manner as described above. As shown in Figure 4, the upregulated heat shock-responsive gene network is dominated by two gene nodes having the highest degree of connectivity relative to the other gene nodes in the network: interleukin-1β (IL1B; extracellular compartment) and the jun oncogene (JUN; nuclear compartment). The results of the subsequent D.A.V.I.D.-based analysis are provided in Table 4 and demonstrate that this upregulated heat shock-responsive gene network is enriched for functional annotations corresponding to molecular chaperone and heat shock protein activity.

**Table 4:** Top 10 functional annotations for two merged networks upregulated in response to heat shock (based on p value).

**Category**	**Term**	**# of genes**	**p-value**
SP_PIR_KEYWORDS	chaperone	11	1.4E-8
GOTERM_BP_ALL	protein folding	14	4.6E-7
SP_PIR_KEYWORDS	heat shock	8	1.2E-7
GOTERM_BP_ALL	response to unfolded protein	9	5.4E-7
GOTERM_BP_ALL	response to protein stimulus	9	5.4E-7
SP_PIR_KEYWORDS	nucleotide-binding	22	3.2E-7
SP_PIR_KEYWORDS	molecular chaperone	6	4.4E-7
GOTERM_MF_ALL	unfolded protein binding	11	4.5E-6
SP_PIR_KEYWORDS	atp-binding	18	6.6E-6
INTERPRO_NAME	chaperonin TCP-1	5	3.8E-4

The analysis is based on the default parameters in D.A.V.I.D. and uses Benjamini-Hochberg correction for multiple testing.

These data demonstrate that the individual upregulated gene lists of LPS- and heat shock-responsive genes correspond to distinct gene networks having unique functional annotations. Complete lists of the respective network genes and a network legend are provided in Additional files 3 to 5.

### Gene network expression corresponding to the downregulated LPS- and heat shock-responsive genes

**Figure 5:**
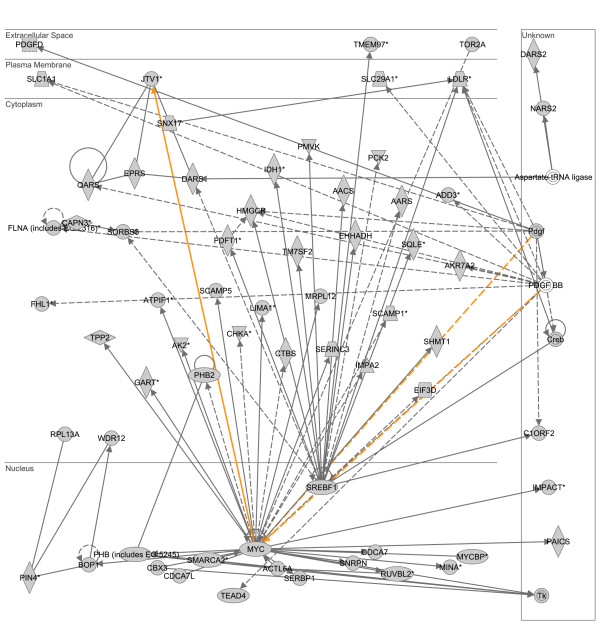
**Merge of two top scoring IPA networks derived from the list of 2,623 downregulated LPS-responsive genes.** The merged network is depicted in the context of cellular compartments. This downregulated LPS-responsive network contains two nodes in the nuclear compartment having a high degree of connectivity to other network genes: v-myc myelocytomatosis viral oncogene homolog (MYC) and sterol regulatory element binding transcription factor 1 (SREBF1). Gold lines indicate connectivity between the two merged networks. See text for network derivation and see Additional file 6 for network gene list and Additional file 4 for network legend.

To derive further biological meaning from the individual lists of downregulated LPS- and heat shock-responsive genes, we conducted an identical analysis as described above based on the IPA application and the D.A.V.I.D. database. The two highest scoring, downregulated LPS-responsive gene networks were merged as depicted in Figure 5. This downregulated LPS-responsive gene network is dominated by two gene nodes, located in the nuclear compartment, and having the highest degree connectivity relative to the other gene nodes in the network: v-myc myelocytomatosis viral oncogene homolog (MYC) and sterol regulatory element binding transcription factor 1 (SREBF1). The results of the subsequent D.A.V.I.D.-based analysis are provided in Table 5 and demonstrate that this downregulated LPS-responsive gene network is enriched for functional annotations corresponding to protein metabolism/synthesis, tRNA-related biochemistry, and sterol metabolism.

**Table 5:** Top 10 functional annotations for two merged networks downregulated in response to LPS (based on p value).

**Category**	**Term**	**# of genes**	**p-value**
SP_PIR_KEYWORDS	aminoacyl-tRNA synthatase	6	1.7E-4
SP_PIR_KEYWORDS	protein biosynthesis	8	5.2E-4
GOTERM_BP_ALL	sterol metabolism	7	6.0E-4
GOTERM_BP_ALL	cellular physiological process	57	7.0E-4
GOTERM_BP_ALL	biosynthesis	21	1.0E-3
GOTERM_MF_ALL	aminoacyl-tRNA ligase activity	6	1.3E-3
GOTERM_MF_ALL	ligase activity, carbon-oxygen bonds	6	1.3E-3
GOTERM_BP_ALL	amino acid activation	6	1.6E-3
GOTERM_BP_ALL	tRNA aminoacylation for protein translation	6	1.6E-3
GOTERM_BP_ALL	tRNA amioacylation	6	1.6E-3

The analysis is based on the default parameters in D.A.V.I.D. and uses Benjamini-Hochberg correction for multiple testing.

**Figure 6:**
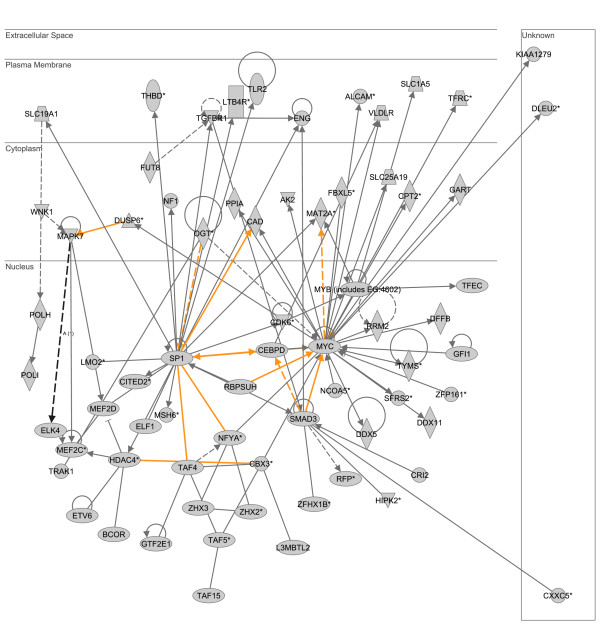
**Merge of two top scoring IPA networks derived from the list of 1,445 downregulated heat shock-responsive genes.** The merged network is depicted in the context of cellular compartments. This downregulated heat shock-responsive network contains two nodes in the nuclear compartment having a high degree of connectivity to other network genes: v-myc myelocytomatosis viral oncogene homolog (MYC) and Sp1 transcription factor (SP1). Gold lines indicate connectivity between the two merged networks. See text for network derivation, and see Additional file 7 for network gene list and Additional file 4 for network legend.

The two highest scoring, downregulated heat shock-responsive gene networks were merged as depicted in Figure 6. This downregulated heat shock-responsive gene network is dominated by two gene nodes, located in the nuclear compartment, and having the highest degree connectivity relative to the other gene nodes in the network: v-myc myelocytomatosis viral oncogene homolog (MYC) and Sp1 transcription factor (SP1). The results of the subsequent D.A.V.I.D.-based analysis are provided in Table 6 and demonstrate that this downregulated heat shock-responsive gene network is enriched for functional annotations corresponding to transcription and nucleic acid metabolism.

**Table 6:** Top 10 functional annotations for two merged networks downregulated in response to heat shock (based on p value).

**Category**	**Term**	**# of genes**	**p-value**
SP_PIR_KEYWORDS	transcription	28	1.7E-11
SP_PIR_KEYWORDS	transcription regulation	28	1.4E-11
SP_PIR_KEYWORDS	nuclear protein	37	4.0E-11
GOTERM_BP_ALL	nucleic acid metabolism	42	1.9E-7
SP_PIR_KEYWORDS	repressor	11	7.9E-7
GOTERM_BP_ALL	regulation, nucleic acid metabolism	32	4.7E-6
GOTERM_BP_ALL	regulation of transcription	31	1.1E-5
GOTERM_BP_ALL	regulation of cellular metabolism	32	9.6E-6
GOTERM_BP_ALL	regulation of cellular process	38	1.2E-5
GOTERM_BP_ALL	regulation of metabolism	32	1.2E-5

The analysis is based on the default parameters in D.A.V.I.D. and uses Benjamini-Hochberg correction for multiple testing.

These data demonstrate that the individual downregulated gene lists of LPS- and heat shock-responsive genes correspond to distinct gene networks having unique functional annotations. Complete lists of the respective network genes and a network legend are provided in Additional files 4, 6, and 7.

### Gene network expression corresponding to the common upregulated LPS- and heat shock-responsive genes

**Figure 7:**
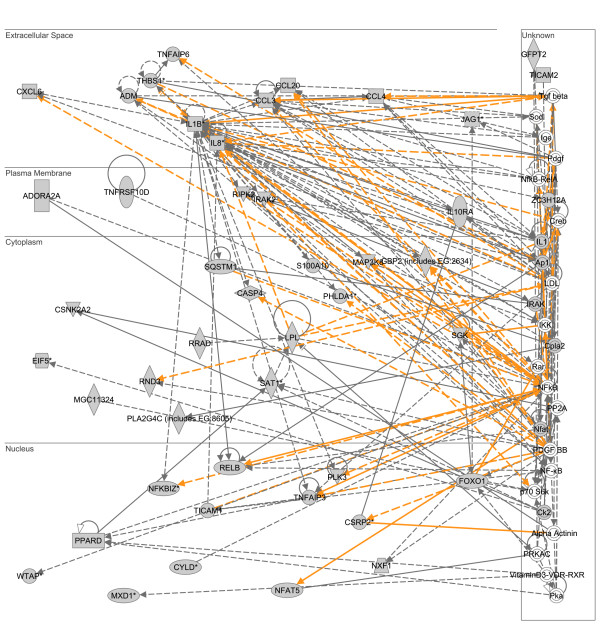
**Merge of two top scoring IPA networks derived from the list of 184 genes common to both the upregulated LPS-responsive gene list and the upregulated heat shock-responsive gene list.** The merged network is depicted in the context of cellular compartments. This upregulated, common LPS/heat shock-responsive network contains two nodes in the extracellular compartment having a high degree of connectivity to other network genes: interleukin-1β (IL1B) and interleukin-8 (IL8). Both the IL1B and IL8 nodes also demonstrate high level connectivity to the NF-κB pathway. Gold lines indicate connectivity between the two merged networks. See text for network derivation, and see Additional file 8 for network gene list and Additional file 4 for network legend.

As previously described (Figure 1), there were 184 genes common to both the upregulated LPS-responsive gene list and the upregulated heat shock-responsive gene list. These 184 common genes were uploaded to the IPA application and the analytical focus was again placed on the presence of gene networks. The two highest scoring, upregulated gene networks corresponding to this 184 common gene list were merged as depicted in Figure 7. This upregulated gene network contains interleukin-1β (IL1B) and interleukin-8 (IL8) as two highly connected gene nodes, located in the extracellular compartment, and also having high level connectivity to the NF-κB pathway. The list of genes corresponding to this upregulated LPS/heat shock-responsive network was subsequently uploaded to an independent database (D.A.V.I.D.) to derive the functional annotations corresponding to the network. The results of the D.A.V.I.D.-based analysis are provided in Table 7 and demonstrate that this upregulated LPS/heat shock-responsive network is enriched for functional annotations corresponding to inflammation and chemokine-related biology. These data demonstrate that the common genes upregulated in response to LPS or heat shock correspond to a gene network associated with inflammation in general, and more specifically with chemokine-related biology. A complete list of the respective network genes and a network legend are provided in Additional files 4 and 8.

**Table 7:** Top 10 functional annotations for two merged networks commonly upregulated in response to both LPS and heat shock (based on p value).

**Category**	**Term**	**# of genes**	**p-value**
GOTERM_BP_ALL	inflammatory response	12	2.9E-8
GOTERM_BP_ALL	response to wounding	14	4.6E-8
GOTERM_BP_ALL	response to external stimulus	14	8.2E-7
INTERPRO_NAME	small chemokine, interleukin-8-like	6	2.7E-4
GOTERM_MF_ALL	protein binding	29	1.5E-4
GOTERM_MF_ALL	chemokine activity	6	8.2E-5
GOTERM_MF_ALL	chemokine receptor binding	6	8.2E-5
GOTERM_BP_ALL	response to stress	16	9.3E-5
SMART_NAME	SCY^1^	6	1.2E-4
SP_PIR_KEYWORDS	chemotaxis	6	3.2E-4

The analysis is based on the default parameters in D.A.V.I.D. and uses Benjamini-Hochberg correction for multiple testing. ^1^Intercrine alpha family (small chemokine C-X-C) (chemokine CXC)

### Gene network expression corresponding to the common downregulated LPS- and heat shock-responsive genes

**Figure 8:**
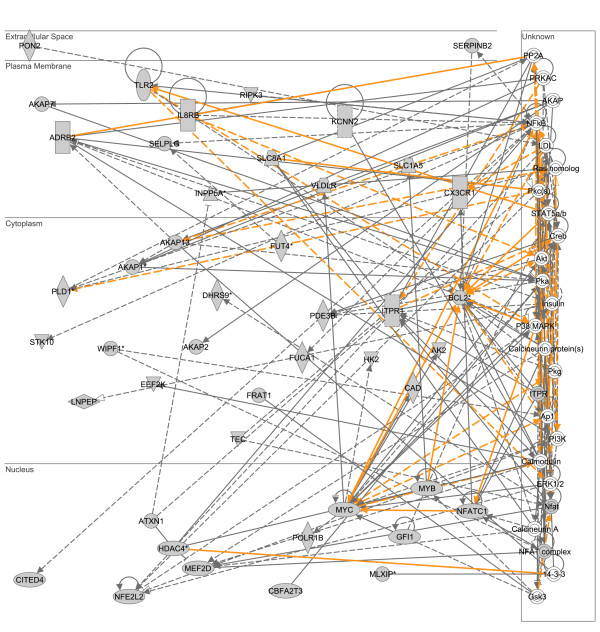
**Merge of two top scoring IPA networks derived from the list of 430 genes common to both the downregulated LPS-responsive gene list and the downregulated heat shock-responsive gene list.** The merged network is depicted in the context of cellular compartments. This downregulated, common LPS/heat shock-responsive network contains two nodes having a high degree of connectivity to other network genes: B-cell CLL/lymphoma 2 (BCL2) in the cytoplasmic compartment and v-myc myelocytomatosis viral oncogene homolog (MYC) in the nuclear compartment. Gold lines indicate connectivity between the two merged networks. See text for network derivation, and see Additional file 8 for network gene list and Additional file 4 for network legend.

As previously described (Figure 2), there were 430 genes common to both the downregulated LPS-responsive gene list and the downregulated heat shock-responsive gene list. These 430 common genes were analyzed in a similar manner to that described above for the common upregulated genes. The two highest scoring, downregulated gene networks corresponding to this 430 common gene list were merged as depicted in Figure 8. This downregulated gene network contains B-cell CLL/lymphoma 2 (BCL2; cytoplasmic compartment) and v-myc myelocytomatosis viral oncogene homolog (MYC; nuclear compartment) as two highly connected gene nodes. The results of the D.A.V.I.D.-based analysis of this network are provided in Table 8 and demonstrate that this downregulated LPS/heat shock-responsive gene network is enriched for functional annotations corresponding to the cellular membrane and phosphorylation. These data demonstrate that the common genes downregulated in response to LPS or heat shock correspond to a gene network associated with the cellular membrane in general, and more specifically with phosphorylation events. A complete list of the respective network genes and a network legend are provided in Additional files 4 and 8.

**Table 8:** Three significant functional annotations for two merged networks commonly downregulated in response to both LPS and heat shock (based on p value).

**Category**	**Term**	**# of genes**	**p-value**
SP_PIR_KEYWORDS	membrane	20	8.2E-3
SP_PIR_KEYWORDS	phosphorylation	14	1.0E-2
SP_PIR_KEYWORDS	kinase	9	3.8E-2

The analysis is based on the default parameters in D.A.V.I.D. and uses Benjamini-Hochberg correction for multiple testing. Unlike the other Tables, Table 8 lists only three functional annotations because all other related annotations did not have significant p values (< 0.05) when subjected to Benjamini-Hochberg correction.

### Top genes expressed in response to LPS or heat shock stress

In order to assess the validity of the data presented above, we extracted the top ten genes expressed in response to LPS or heat shock stress (based on fold induction over control conditions). The majority of the top ten genes expressed in response to LPS stress (Table 9) are well established LPS-responsive genes. Similarly, the top ten genes expressed in response to heat shock stress (Table 10) are well established heat shock-responsive genes. These data demonstrate consistency with known LPS- and heat shock-related biology, thus adding an important level of validity to the data presented in the previous sections.

**Table 9:** Top 10 genes (based on fold change versus control cells) expressed in response to LPS stress.

**Description**	**Gene Symbol**	**Fold Change**
tumor necrosis factor, alpha-induced protein 6	TNFAIP6	1,696
chemokine (C-C motif) ligand 20 *Alias: MIP-3α*	CCL20	1,036
chemokine (C-C motif) ligand 4 *Alias: MIP-1β*	CCL4	749
insulin-like growth factor binding protein 3	IGFBP3	519
interleukin 1, beta	IL1B	446
BCL2-related protein A1	BCL2A1	399
interleukin 8	IL8	365
lysosomal-associated membrane protein 3 *Alias: CD208*	LAMP3	359
Epstein-Barr virus induced gene 3 *Alias: Interleukin-27*	EBI3	315
interleukin receptor 7	IL7R	205

**Table 10:** Top 10 genes (based on fold change versus control cells) expressed in response to heat shock stress.

**Description**	**Gene Symbol**	**Fold Change**
heat shock 70 kDa protein 6	HSPA6	3,580
heat shock 70 kDa protein 1B	HSPA1B	1,526
immediate early response 5	IER5	230
collagen, type I, alpha 1	COL1A1	183
heat shock 70 kDa protein 1A	HSPA1A	170
Procollagen-proline, 2-oxoglutarate 4-dioxygenase (proline 4-hydroxylase), alpha polypeptide II	P4HA2	118
BCL2-associated athanogene 3 *Alias: BAG-family molecular chaperone regulator-3*	BAG3	111
DnaJ (Hsp40) homolog, subfamily B, member 4 *Alias: DnaJ-like heat shock protein 40*	DNAJB4	106
dual specificity phosphatase 1 *Alias: MKP-1*	DUSP1	96
v-jun sarcoma virus 17 oncogene homolog *Alias: c-jun*	JUN	82

## Discussion

We have characterized the genome-level responses of THP-1 cells (a model of peripheral blood mononuclear cells) following stimulation with one of two canonical stress/danger signals at a single time point. While previous high throughput-centered studies have focused on either LPS [7-12] or heat shock stress alone [28-34], to our knowledge the current work represents the first direct comparison of LPS and heat shock stress based on the same analytical approach. Importantly, the analytical approach employs three independent genomic expression discovery databases (i.e. PANTHER, IPA, and D.A.V.I.D.), thus adding to the trustworthiness of the derived functional annotations and networks.

The global view (Venn diagrams) of the genes differentially regulated in response to LPS or heat shock stress indicates that each stress signal regulates (increased or decreased expression) a relatively unique group of genes. Among the upregulated genes, > 85% of the genes were uniquely expressed in response to either LPS or heat shock stimulation. Among the downregulated genes, > 69% of the genes were uniquely repressed in response to either LPS or heat shock stimulation. Thus, the mononuclear cell response to LPS stress is generally divergent compared to that of heat shock stress.

The divergence between the LPS response and the heat shock response is evident by the functional annotations derived for the respective individual lists of differentially regulated genes. The functional annotations derived for the upregulated LPS-responsive genes were predominantly in the areas of inflammation and immunity. This observation is consistent with the well known role of LPS as the major pathogen associated molecular pattern of gram negative bacteria, and the well known role of peripheral blood mononuclear cells as sentinels of the innate immune system [35]. In contrast, the functional annotations derived for the upregulated heat shock-responsive genes were predominantly in the areas of heat shock proteins, protein unfolding, and molecular chaperones. This observation is also consistent with the existing literature surrounding the heat shock response and heat shock proteins [2,36].

The assertion of divergence is further supported by the functional annotations derived for the individual lists of differentially downregulated genes. The functional annotations derived for the downregulated LPS-responsive genes were predominantly in the areas of protein biosynthesis and sterol metabolism. These observations are indirectly supported by reports documenting LPS-mediated alterations of protein and lipid metabolism [37-42]. In contrast, the functional annotations derived for the downregulated heat shock-responsive genes were predominantly in the areas of nucleic acid metabolism and regulation of transcription. These observations are consistent with the aforementioned paradigm in which the heat shock response is characterized by a re-prioritization of gene expression toward heat shock protein transcription while non-heat shock protein gene transcription is globally repressed [4-6]. This paradigm is likely to be dependent on the time frame after heat shock. For example, global transcriptional repression may be most evident immediately following heat shock. However, the relatively large number of "non-heat shock protein" genes expressed four hours after THP-1 cells were subjected to heat shock indicates that the concept of global transcriptional repression following heat shock may be an oversimplification. The assertion of oversimplification is further supported by the work of previous investigators employing microarray technology to study the heat shock response of mammalian and non-mammalian systems [28-34]. These previous investigations were also notable for the expression of "non-heat shock protein" genes in response to heat shock.

In the context of our previous studies, several of the genes upregulated in response to heat shock or LPS warrant further discussion. Dual specificity phosphatase 1 (DUSP1, a.k.a. MKP-1) was substantially increased in THP-1 cells subjected to heat shock. MKP-1 is a dual specific phosphatase serving an important counter-regulatory function in the MAP kinase signaling pathway [15,43,44]. We previously demonstrated that MKP-1 is a heat shock-responsive gene in cultured murine macrophages and that heat shock-mediated regulation of the MKP-1 gene is dependent on both transcriptional and post-transcriptional mechanisms [14]. Sonna et al also reported MKP1/DUSP1 expression in primary human mononuclear cells exposed to heat shock [33].

Several laboratories, including our own, have previously reported that heat shock inhibits activation of the NF-κB signaling pathway [3,13,16-18], [45-47]. In this regard it was interesting to note that nuclear factor of kappa light polypeptide gene enhancer in B-cells inhibitor zeta (NFKBIZ), NFKB inhibitor interacting Ras-like 2 (NKIRAS2), and NF-kappaB repressing factor (NKRF) were coordinately upregulated in THP-1 cells subjected to heat shock (see *Supplementary Data*). All three of these genes appear to play important negative regulatory roles in the NF-κB signaling pathway. NFKBIZ is a member of the ankryin-repeat family of the IκB group of proteins that bind and retain NF-κB in the cytoplasm in an inactive state [48]. NKIRAS2 is a Ras protein subclass reported to regulate the degradation of IκB [49]. Finally, NKRF has been reported to interact with specific negative regulatory elements that mediate transcriptional repression of NF-κB responsive genes [50] and has been previously reported to be expressed in primary human mononuclear cells exposed to heat shock [33].

LPS is a key pathogen associated molecular pattern that is well known to play an important role in the pathobiology of human septic shock [1,35]. In separate translational studies we have been conducting genome-wide expression profiling in children with septic shock [21,22,51]. One of the novel findings generated from this translational program is that pediatric septic shock is characterized by large scale downregulation of genes having zinc-related functional annotations. In keeping with this observation, we have also reported that nonsurvivors of pediatric septic shock have abnormally low serum zinc concentrations [21]. In light of these findings and the important role of LPS in septic shock, we searched the list of genes downregulated in THP-1 cells subjected to LPS stress for enrichment of zinc-related functional annotations. We found between 23 and 28 genes having functional annotations for the terms "zinc", "zinc ion binding", "zinc-finger", or "metal-binding" (data not shown, analysis based on the D.A.V.I.D. database). Thus, in keeping with our clinical, translational studies we have validated *in vitro *that LPS suppresses the expression of genes that either depend on zinc homeostasis or directly participate in zinc homeostasis.

The most novel information derived from the current data involves the list of genes commonly upregulated in response to LPS and heat shock stress. There were 184 genes common to both the upregulated LPS gene list and the upregulated heat shock-responsive gene list. This relatively limited number of genes corresponded to gene networks having functional annotations related to inflammation in general, and more specifically to chemokine-related biology. Thus, the response to heat shock stress is not completely divergent from that of the LPS response in that it shares a group of coordinately regulated genes related to chemokine signaling, a heretofore unreported observation.

## Conclusion

We have directly compared the genomic response of THP-1 cells subjected to LPS stress or heat shock stress. The global gene expression patterns demonstrate that the two responses are predominantly divergent. The response to heat shock, however, is not necessarily characterized by global downregulation of non-heat shock protein genes. In fact, a large number of "non-heat shock protein" genes are expressed in response to heat shock stress and a number of these genes have overlap with inflammation-associated gene expression patterns classically attributed to LPS stress. Thus, the heat shock response and the LPS response are not necessarily mutually exclusive. Finally, the data generated from these experiments can serve as a valuable reference database for investigators in the field of stress/danger signaling.

## Authors' contributions

HRW conceived and directed the overall study, conducted analyses, and drafted the initial manuscript. KO conducted all microarray experiments. BS assisted with data analysis and bioinformatics. All authors proof read the manuscript and agreed with its submission for review.

## Supplementary Material

Additional file 1 3968 genes regulate in lps treated thp1 cells.xls, list of genes regulated in response to LPS alone. Click here for file

Additional file 2 2921 genes regulate in heat shocked thp1 cells.xls, list of genes regulated in response to heat shock alone. Click here for file

Additional file 3 merged uregulated lps network.xls, list of genes corresponding to two merged, upregulated networks in response to LPS alone and shown in Figure 3. Click here for file

Additional file 4 network legend.pdf, legend for networks depicted in Figures 2, 3, and 5 to 8. Click here for file

Additional file 5 merged upregulated heat shock network.xls, list of genes corresponding to two merged, upregulated networks in response to heat shock alone and shown in Figure 4. Click here for file

Additional file 6 merged downregulated LPS network.xls, list of genes corresponding to two merged, downregulated networks in response to LPS alone and shown in Figure 5. Click here for file

Additional file 7 merged downregulated heat shock network.xls, list of genes corresponding to two merged, downregulated networks in response to heat shock alone and shown in Figure 6. Click here for file

Additional file 8 genes common to both lps and heat shock.xls, list of genes commonly regulated in THP1 cells treated with either LPS or heat shock. Click here for file
