# Supplementary material for: Divergence of canonical danger signals: The genome-level expression patterns of human mononuclear cells subjected to heat shock or lipopolysaccharide
Source: BMC Immunol. 2008 May 30;9:24. doi: 10.1186/1471-2172-9-24 (PMC2430197; doi:10.1186/1471-2172-9-24)

NETWORK LEGEND

Gold lines indicate connectivity between the two merged networks.

An arrow pointing from A to B signifies different actions for different circumstances, as described below:

For signaling pathways:
An arrow pointing from A to B signifies that A causes B to be activated (includes any direct interaction: e.g. binding, phosphorylation, dephosphorylation, etc).

For metabolic pathways:
An arrow pointing from A to B signifies that B is produced from A.

For ligands/receptors:
An arrow pointing from a ligand to a receptor signifies that the ligand binds the receptor and subsequently leads to activation of the receptor. This binding event does not necessarily directly activate the receptor; activation of the receptor could be caused by events secondary to the ligand/receptor binding event.

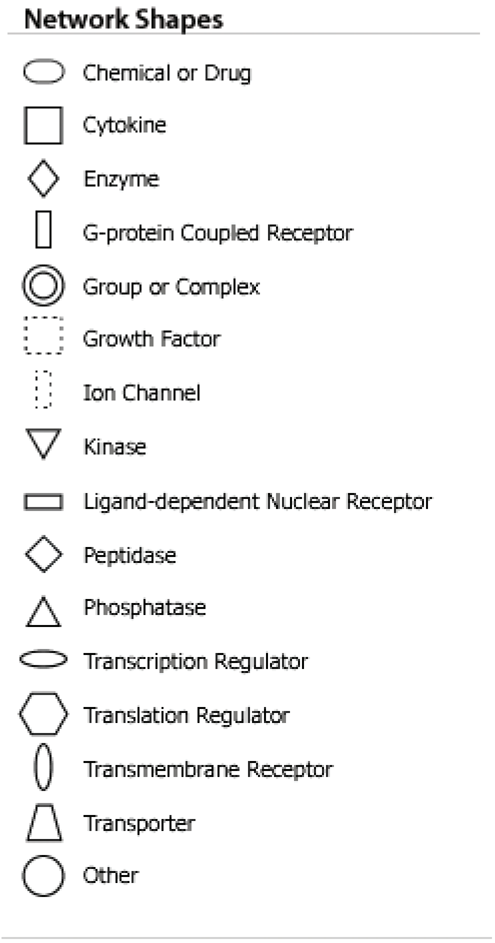

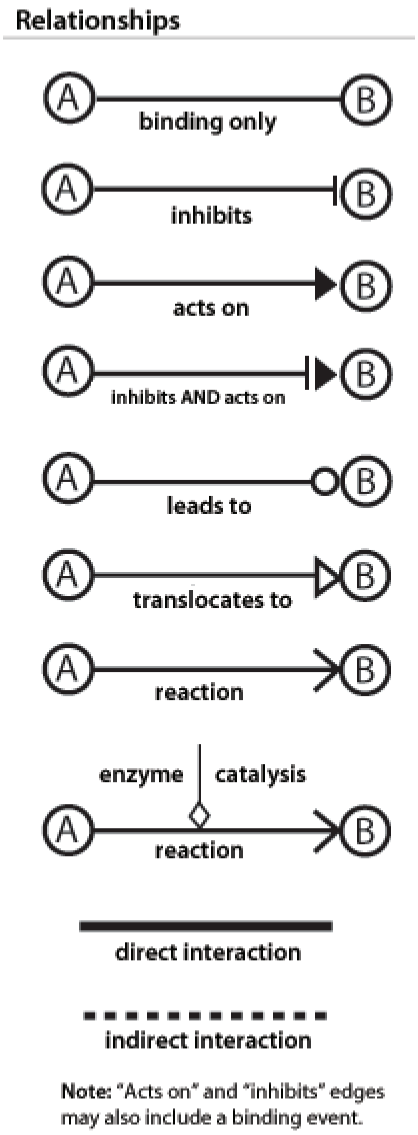

Supplement: Additional file 4 — network legend.pdf, legend for networks depicted in Figures 2, 3, and 5 to 8. [file 1471-2172-9-24-S4.pdf]
